# Laterite Integrated Persulfate Based Advanced Oxidation and Biological Treatment for Textile Industrial Effluent Remediation: Optimization and Field Application

**DOI:** 10.1155/abb/9325665

**Published:** 2025-02-19

**Authors:** Davuluri Syam Babu, Kunamineni Vijay, Shaik Shakira, Venkatasai Sumasri Mallemkondu, Puspita Barik, Chandrasekhar Kuppam, Vallayyachari Kommoju, Indira Mikkili, Adamu Mulatu, Pinapala Chanikya, M. V. Raju

**Affiliations:** ^1^Department of Bio Technology, Vignan's Foundation for Science, Technology and Research, Vadlamudi, Guntur 522213, Andhra Pradesh, India; ^2^Department of Civil Engineering, Vishnu Institute of Technology, Bhimavaram 534202, Andhra Pradesh, India; ^3^Environmental Monitoring Solutions, Acoem Ecotech Industries Pvt., Ltd., Pithampur 454775, Madhya Pradesh, India; ^4^Civil Engineering Department, Wollega University, Nekemte, Ethiopia; ^5^Department of Civil Engineering, Vignan's Foundation for Science, Technology and Research, Vadlamudi, Guntur 522213, Andhra Pradesh, India

**Keywords:** biological treatment, hybrid process, persulfate oxidation process, sulfate radical, textile effluent

## Abstract

This study investigated a combined approach of a persulfate-based advanced oxidation process (AOP) followed by biological treatment of a textile industrial effluent. The effluent from the textile industry is primarily composed of various dyes in varying concentrations, resulting in high chemical oxygen demand (COD) and biological oxygen demand (BOD). The model pollutant rhodamine B (RhB) was used in the optimization studies. During the persulfate oxidation process (PSO), persulfate activation is required to generate sulfate radicals (SO_4_^•−^). Raw laterite soil was used as a catalyst for the treatment of RhB in batch studies, and it was able to reduce the dye concentration by about 20% in 60 min of operation, with initial RhB concentrations of 150 mg L^−1^ and persulfate concentrations of 200 mg L^−1^. Furthermore, alkali-treated laterite soil (ATLS) was used as a catalyst, achieving 57%–60% removal in 60 min at pH 3 and complete removal after 72 h of biological treatment. Furthermore, the optimized conditions were tested on real field waters to determine efficiency, and it was observed that the PSO removed approximately 45% of COD, with further biological treatment for 72 h increasing the removal efficiency to 64%. All other parameters of water quality were reduced by more than 60%.

## 1. Introduction

The dying of textiles is the world's second-largest source of water pollution, and the fashion sector alone is responsible for producing 20% of all wastewater globally [1, 2]. This is because companies that manufacture textiles require significant quantities of water, and the wastewater produced, as a result, creates significantly contaminated output [1, 3]. Textile wastewater contains various contaminants, including dyes, chemicals, and suspended solids, which can have harmful effects on aquatic life and human health [4, 5]. Proper treatment and management of textile wastewater are essential to protect the environment and ensure sustainable textile production. The textile and clothing industries used approximately 79 billion cubic meters of water [6]. The quantity of water required varies based on the specific type of cotton, manufacturing methods, and the geographical location of cotton fields and factories. To produce a single t-shirt, approximately 2700 L of water are required, equivalent to 5.9% of an individual's annual drinking water intake. This highlights the significant water usage involved in the garment production process [7]. Thus, the textile sector should minimize the water usage and improve water management [2]. The synthetic microfibers released while manufacturing cloths and washing contribute significantly to the problem of microplastic pollution in the ocean [8]. According to studies, microfibers from synthetic textiles such as polyester and nylon are released into the ocean at an annual rate of roughly 0.5 million tones, accounting for about 35% of all primary microplastics discharged into the environment [9].

Conventional physicochemical processes for textile wastewater treatment typically involve a combination of unit operations such as membrane filtration [10], coagulation [11], adsorption [12], and chemical oxidation processes. Coagulation involves the addition of a coagulant to destabilize and agglomerate suspended particles. Flocculation promotes the formation of larger particles, which are easier to separate by sedimentation or filtration. Numerous studies have explored the treatment of textile wastewater using coagulation processes. Adachi et al. [13] achieved 69% decolorization and 90% turbidity removal using natural bio-coagulants and bio-flocculants. Another study compared chemical coagulation (CC) and electrocoagulation (EC), finding that EC achieved 63.05% chemical oxygen demand (COD) reduction, 99.07% color removal, and 96.31% turbidity removal, while CC achieved 54.02% COD reduction, 96.21% color removal, and 93.7% turbidity removal [14]. Although coagulation processes are cost-effective and biodegradable, they have limitations such as higher operating costs for EC, sludge generation, and the need for parameter optimization like pH and coagulant concentration to achieve optimal results [13, 14]. Adsorption processes also show promise in treating textile wastewater. Liugė and Paliulis [15] reported up to 97% dye removal efficiency using aerogels. Moreover, unmodified, H_2_SO_4_ modified, and chitosan-coated banana fibers have demonstrated significant removal efficiencies for dyes and other contaminants, with chitosan-coated fibers performing the best [16]. While adsorption methods are effective and cost-efficient, they can generate toxic sludge and require precise optimization of dosages, contact times, and pH levels [17]. Membrane processes such as nanofiltration and reverse osmosis have been extensively studied for their ability to treat textile wastewater. Rathinam et al. [18] found that these methods can remove 90%–99% of pollutants. However, membrane fouling, energy intensity, and operational costs are significant drawbacks. Despite these issues, membrane processes remain promising due to their high removal rates and ability to produce high-quality effluent [18]. Other studies have highlighted the effectiveness of various membrane processes. Hatimi et al. [19] reported over 99% turbidity and COD removal using ultrafiltration membranes synthesized from pyrrhotite ash and natural clay. Similarly, Maheshwari, Agarwal, and Ghosh [20] demonstrated a 99.7% removal of Congo red dye using a polyamide-based nanofiltration membrane. These studies underscore the potential of membrane processes in addressing textile wastewater treatment challenges [21]. EC processes have also shown promising results. Akkaya and Üçgül [22] achieved up to 92% COD removal and 95% color removal from textile effluents. EC offers advantages such as low energy requirements, minimal sludge generation, and ease of setup [23]. However, EC faces challenges like periodic electrode maintenance, potential scaling issues, and sludge disposal, which researchers suggest could be mitigated through innovative methods such as converting sludge into brick or ceramic materials [24]. These processes effectively remove suspended solids, organic matter, and some pollutants, but they may require large capital and operating costs, produce sludge, and require extensive chemical handling and storage. However, conventional physicochemical processes for the treatment of textile wastewater are limited in their ability to remove or degrade certain pollutants. These pollutants include persistent organic pollutants, refractory organic compounds, and toxic heavy metals, as well as membrane fouling [25], sludge bioaccumulation, and secondary pollutant formation. In addition, conventional physicochemical processes for textile wastewater treatment also contribute to the formation of secondary pollutants [5]. To overcome this limitation, the oxidation of these pollutants is necessary before their removal or degradation. Oxidation processes, such as advanced oxidation processes (AOPs) or biological oxidation, can break down or transform these pollutants into less harmful forms, making them more amenable to conventional treatment processes. AOPs, which include methods such as ozonation, photocatalysis, and Fenton's reagent, use highly reactive oxidants to generate hydroxyl radicals (HO^•^) that can degrade a wide range of pollutants. Biological oxidation, which involves the use of microorganisms to degrade pollutants, is effective in treating biodegradable organic pollutants. By combining oxidation processes with conventional physicochemical processes, textile wastewater treatment can be made more efficient and sustainable, leading to cleaner water and a healthier environment. The oxidation of the pollutants is necessary before their removal or degradation. Recently, the application of various AOPs have been used for various industrial effluents, namely, textile industrial wastewater [5, 26, 27], composite wastewater [28–31], and pharmaceutical wastewater [32]. Integrated steel plant wastewater [33] and groundwater contaminated with arsenic [34–37] are demonstrating that the prior oxidation process shows a greater effect on total removal efficiency. Our earlier research [5] investigated the electrochemical persulfate oxidation process (PSO) as a pretreatment for treating textile wastewater before EC to remove the oxidized contaminants. Electrochemical methods were used to activate persulfate to generate sulfate radicals (SO_4_^•−^); however, the drawback of this method is that iron electrodes and an external electrical supply are required for the activation. Additionally, it has been shown that a significant volume of sludge is produced after treatment. Because of its toxicity, it is well known that managing sludge is an additional challenge. In order to get over these limitations, laterite soil and alkali-treated modified laterite soil were investigated for the activation of persulfate in this work. The material was readily available, environmentally friendly, and affordable. To further treat the wastewater from the textile industry, biological treatment, which is anaerobic microorganisms used for the treatment of textile industry effluent, was investigated as a secondary treatment to degrade the oxidized contaminants.

## 2. Material and Methods

### 2.1. Chemicals and Materials

All the chemicals utilized in this procedure were of analytical grade and were employed without further purification. Dextrose sourced from Sigma–Aldrich, yeast extract from Sigma–Aldrich, rhodamine B (RhB) dye from Sigma–Aldrich, magnesium sulfate heptahydrate (MgSO_4_·7H_2_O) from Sigma–Aldrich, calcium chloride dihydrate (CaCl_2_·2H_2_O) from Sigma–Aldrich, ferric chloride hexahydrate (FeCl_3_·6H_2_O) from LabChem, copper (II) chloride dihydrate (CuCl_2_) from Sigma–Aldrich, zinc chloride (ZnCl_2_) from LabChem, nickel (II) chloride hexahydrate (NiCl_2_·6H_2_O) from Sigma–Aldrich, and cobalt chloride (CoCl_2_) from Sigma–Aldrich were procured from local suppliers. Reagents were prepared using distilled water.

### 2.2. Spectroscopic Analysis

Shimaduz 1700 UV–Vis spectrometer was used to investigate the between 190 and 800 nm spectra. The residual RhB concentration was assessed at an absorbance of 554 nm, corresponding to the visible spectrum peak wavelength for RhB.

### 2.3. Acclimatization of Microorganisms From Cow Dung

An anaerobic batch reactor of 1 L capacity was used for the biomass acclimation process. Cow dung slurry was used as the biomass source, and tap water was diluted with it at a ratio of 1:10 (slurry: water). During this procedure, a heterogeneous culture was developed in an anaerobic reactor with synthetic feed containing dextrose (1000 mg L^−1^), yeast extract (50 mg L^−1^), trace metal solution (1 mg L^−1^), and phosphate buffer (1 mg L^−1^). The trace metal solution contains the following amounts of each element: MgSO_4_·7H_2_O (10,000 mgL^−1^), CaCl_2_·2H_2_O (10,000 mg L^−1^), FeCl_3_·6H_2_O (5000 mg L^−1^), CuCl_2_, ZnCl_2_, NiCl_2_·6H_2_O (1000 mg L^−1^), and CoCl_2_ (500 mg L^−1^). Sponge cubes measuring 1 cm × 1 cm × 1 cm as a supportive medium for biomass growth were used. Eight grams of sponge were put into a reactor with a 1-L capacity and anaerobic conditions were maintained. The temperature of the reactor was kept constant at 30 ± 2.0°C and the pH at 7.0 ± 0.5 was maintained by adding 0.1 N HCl/NaHCO_3_.

### 2.4. Biological Degradation of Rhodamine-B

After acclimatization in the attached growth anaerobic batch reactor, a conical flask with a rubber cork and 1500 mL capacity, ideal for the original reactor and having the facility of inlet and outlet for feeding microorganisms and collecting the sample at regular intervals, was started. In a conical flask reactor with a capacity of 60%–80%, 8.0 g sponge cubes (400 sponge pieces) were introduced. These micro reactors were run under conditions identical to the original parent batch reactor to test their efficacy on dye removal at various beginning dye concentrations. In this initial study, the dye concentration varied from lower to higher (1–250 mg L^−1^). The samples were collected at regular intervals (24 h) and analyzed using spectroscopy.

### 2.5. Laterite Soil and Alkali Modified Laterite Soil as a Catalyst

The laterite soil was collected from a quarry adjacent to the institution (Vignan Foundation for Science, Technology, and Research, Vadlamudi, Guntur, Andhra Pradesh, India). Four samples were selected at random from the area for standard soil sampling. The soil was collected at a depth of 15 cm below the surface. The collected soil samples were combined to make one composite soil sample. Using a mortar and pestle, the laterite soil was reduced to fine powder, which was then sieved using sieves with varying 1–2 mm mesh sizes. The soil samples were cleaned with distilled water to remove any organic material before being dried in the sun to create fine powders. The catalyst for raw laterite was used directly. One hundred grams of fine laterite particles were treated with 1 L of a 10 M NaOH solution to prepare modified laterite. The use of a magnetic stirrer contributed to the success of the mixing process. After the mixture was thoroughly combined, the solid particles were rinsed in distilled water until the pH level of the supernatant reached a neutral state. After that, the modified laterite was segregated and air-dried in a hot air oven until it was dry [28].

### 2.6. Persulfate Oxidation Process

Batch experiments were performed in a 1-L glass beaker. To ensure uniformity in the wastewater, a magnetic stirrer (Remi, India) and a 5 cm magnetic bead were employed for continuous mixing at 150 rpm. Increased mixing speeds are hypothesized to disrupt material agglomeration, thereby, effectively improving processability [38]. The current study abides with hydrodynamics of system of Babu et al. [36] modeled based on OpenFOAM. The reactor system attained uniform speed with vigorous and thorough mixing owing to the hydrodynamics of the system with magnetic stirrer propelling the mixing mechanism. In the batch experiments, magnetic bead placed in glass beaker on a stirrer enables uniform and effective mixing of catalyst and reagent based on concept multiple reference frame (MRF) [36]. Initially, experiments were conducted with varying PS concentrations of 50, 100, 150, 200, and 250 mg L^−1^. Subsequently, the raw laterite soil and catalyst doses of 50, 100, 150, and 200 mg L^−1^ were investigated for further activation. The pH of the solution was adjusted using 0.4 N sulfuric acid or 0.4 N sodium hydroxide solutions. Regular samples were analyzed using spectroscopy to measure residual dye concentrations, evaluating the effectiveness of the treatment process.

### 2.7. Hybrid Process

The hybrid process involves an initial persulfate oxidation treatment followed by a biological treatment. The PSO was conducted for 60 min, during which samples were collected every 15 min to analyze the residual dye concentration. After the 60-min persulfate treatment, the wastewater underwent biological treatment in a well-established and acclimated microbial culture reactor for 23 h. The combined hybrid process, thus, spanned a total of 24 h. Posttreatment, samples were subjected to centrifugation to eliminate cellular debris, and the supernatant containing the residual dye was analyzed using spectroscopy to determine its concentration.

## 3. Results and Discussion

### 3.1. Biological Treatment of Rhodamine-B

**Figure 1:**
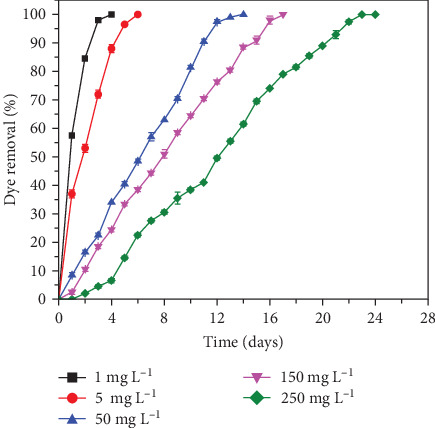
Biological degradation of RhB. (Experimental conditions: RhB: 1, 5, 50, 150, and 250 mg L^−1^; pH: 7.00 ± 0.5; and temperature: 30 ± 2.0°C).

The initial experiments performed by varying the dye concentration from 1 to 250 mg L^−1^ under consistent conditions similar to those used during the acclimation phase. The anaerobic biological process effectively degraded RhB at 1 mg L^−1^ within 4days (as depicted in the figure using average values). The time required for complete dye removal increased with higher initial dye concentrations. Specifically, RhB degradation was achieved in 5, 14, 17, and 23 days for initial concentrations of 5, 50, 150, and 250 mg L^−1^, respectively. Lower concentrations exhibited faster degradation, while higher concentrations, particularly 150 and 250 mg L^−1^, required more than 15 days for complete removal (Figure 1). Consequently, the 150 mg L^−1^ concentration was chosen for further experimentation. Similarly, Abu Ghunmi and Jamrah [39] investigated the effectiveness of using a sequencing batch reactor (SBR) for treating textile wastewater characterized by high temperatures (averaging 60°C), alkaline pH levels (averaging 9.5), and substantial pollutant concentrations, including BOD_5_ (600 mg L^−1^) and COD (1000 mg L^−1^). Their SBR experiments achieved up to 95% removal of soluble COD (SCOD) within 22–25 h, maintained by optimal substrate to microorganism ratios (*S*_0_/*X*_0_) between 0.13 and 0.5 on a biological oxygen demand (BOD)/MLSS basis. The highest removal of BOD reached 91% at an *S*_0_/*X*_0_ ratio of 0.16. The effectiveness of the SBR process was evident in the removal of dissolved solids (8%–48%) and suspended solids (0.4%–28%). Moreover, Araújo et al. [40] examined biological treatment for textile wastewater with elevated salinity (up to 12.6 gkg^−1^) and surfactant (up to 5.9 mg L^−1^ of (linear alkylbenzene sulfonate (LAS))), targeting color (up to 652 mg Pt-Co L^−1^), and sulfate (up to 1568.6 mg SO_4_^2−^·L^−1^) removal. It was observed that supplementation with ethanol (ET) and molasses in anaerobic batch reactors significantly enhanced sulfate reduction (23% without vs. 87% with supplementation), aiding in reactor recovery post higher salinity impact. Aromatic amine removal by the aerobic reactor was effective below 1.0 mg LAS·L^−1^, but was hindered at 5.9 mg LAS·L^−1^, highlighting the potential of biodegradable organic matter supplementation to mitigate wastewater variability. These systems utilize microbial communities to break down contaminants through oxidation–reduction reactions, generating clean energy in the form of bioelectricity [41]. Biological treatment processes have shown promising results in the oxidation of dye wastewater. Studies have demonstrated that aerobic biological treatment can effectively reduce COD levels in dye effluents, with specific rates of organic pollutant oxidation averaging 12.4 mg/(gh) for COD and 9 mg/(gh) for BOD [42]. Additionally, the combined approach of persulfate oxidation followed by biological treatment has proven successful in reducing dye concentrations by approximately 20% in batch studies, with further removal efficiencies reaching up to 64% after 72 h of treatment [43]. Furthermore, the utilization of bacterial consortia in conjunction with photocatalytic processes has shown significant dye degradation rates, with the best-performing combination achieving a pollutant degradation rate of 64.62% [44]. These findings highlight the efficacy of biological treatment methods in the oxidation of dye wastewater, showcasing their potential for sustainable and efficient wastewater treatment processes. While biological treatment shows promise for textile wastewater remediation, the complex nature of pollutants necessitates the integration of advanced oxidation techniques, such as ozone-based processes (AOP) coupled with nanobubble generators, to achieve substantial removal of pollutants like COD and ammonia content [45]. Integration with technologies like nanofiltration and UV/hydrogen peroxide (H_2_O_2_) treatments enhances removal efficiencies, making water suitable for reuse in textile industry processes [46, 47]. These advancements are essential for enhancing the efficiency and sustainability of textile wastewater treatment, ensuring complete removal of contaminants for environmental safety.

### 3.2. PSO

**Figure 2:**
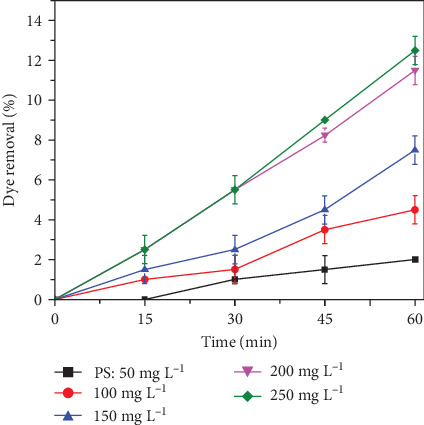
Effect of persulfate dose on RhB degradation. (Experimental conditions: RhB: 150 mg L^−1^; persulfate dose 50, 100, 150, 200, and 250 mg L^−1^; and pH: 7.00 ± 0.5).

Initial experiments were conducted to investigate the effect of PS dosage on dye decolourization. The PS dose was varied from 50, 100, 150, and 200 mg L^−1^ to 250 mg L^−1^, with an initial dye concentration of 150 mg L^−1^ at pH 7.00 ± 0.5 for 60 min. Samples were collected at 15 min intervals up to 60 min to measure the residual dye concentration. The decolorization of RhB was achieved with the addition of PS, as shown in Figure 2, which illustrates the results at different PS dosages. The decolorization efficiency increased with higher PS doses. Persulfate acts as a direct oxidant, effectively oxidizing RhB. Although persulfate alone can oxidize pollutants to some extent [48, 49], its effectiveness in degrading high organic content is limited [5, 50]. This can be attributed to the generation of peroxides and oxygen as expressed in Equations (1) and (2), respectively.

(1) $$\begin{array}{c} S_{2}O_{8}^{2-}+{\text{H}}_{2}\text{O}\to {\text{HSO}}_{4}^{-}+0.5{\text{O}}_{2}. \end{array}$$

(2) $$\begin{array}{c} S_{2}O_{8}^{2-}+{\text{H}}_{2}\text{O}\to 2{\text{HSO}}_{4}^{-}+0.5{\text{H}}_{2}O_{2}. \end{array}$$

Previous studies using electrochemical persulfate oxidation to treat textile wastewater [5] and arsenic contaminated water [35] reported similar findings, noting that higher persulfate concentrations could increase side reactions [51]. The effect of persulfate dose on dye removal has been extensively studied. Research indicates that persulfate activation is crucial for generating SO_4_^•−^ that aid in dye degradation [52, 53]. The persulfate dose of 200 mg L^−1^ reduced RhB concentration by about 20% in 60 min [54]. Additionally, a study using 1g L^−1^ persulfate with methylene blue achieved approximately 94% discoloration and 45.7% total organic carbon (TOC) reduction after 180 min [55]. In another study, a 4 mM persulfate dose optimally removed 97.4% of sunset yellow dye after 120 min. The pH of the solution is crucial for the efficiency of persulfate based dye removal processes. Optimal pH ranges for high discoloration rates have been identified, such as 4.0–7.0 for methylene blue [53] and acidic to neutral values for AY17 dye when using a polyaluminum chloride (PAC) catalyst [54]. For methylene blue removal via copper oxide-activated persulfate, a pH of 7.0–9.0 was optimal, achieving over 90% degradation [56]. On the other hand, treating reactive black 5 dye with lanthanum iron oxide under UV-A irradiation was effective only at pH of 4 or lower [57]. Thus, maintaining appropriate pH levels is essential for enhancing persulfate-based dye removal efficiency. The decolorization of RhB was high at both 200 and 250 mg L^−1^ PS, with similar results in both cases. Therefore, 200 mg L^−1^ PS was used as the optimal dose for subsequent experiments.

### 3.3. Activation of Persulfate Using Laterite Soil and Modified Laterite Soil

Activation of PS is essential for the effective generation of SO_4_^•−^ in the aqueous medium [35]. Various activation methods like heat activation [58], metal ions [59], ultrasound, ultraviolet irradiation, microwave radiation [60], activated carbon, H_2_O_2_, and electrochemical activation generate SO_4_^•−^ and ^•^OH radicals from PS and peroxi mono sulfates (PMS) [61]. However, the higher energy consumption in the thermal activation method, the costlier microwave activation, and the higher chemical requirement make them practically unacceptable. The utilization of naturally available resources, hence, is used for the activation of PS.

**Figure 3:**
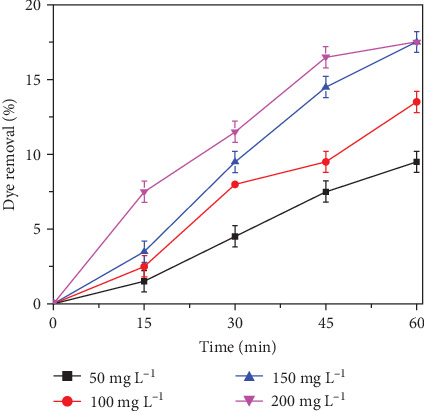
Effect of raw laterite soil dosage as a catalyst on persulfate activation for the decolorization of RhB (Experimental conditions: RhB: 150 mg L^−1^, persulfate dose: 200 mg L^−1^, pH: 7.00 ± 0.5).

Laterite soil contains iron (Fe_2_O_3_) content ranging from 16% to 67% and is accessible for extraction [62]. In wastewater decontamination studies, iron (Fe) is utilized as a catalysts [63], Fenton reagent [64], and activator (persulfate activation by heavy metals) [5]. The current study focuses on the potential application of raw and modified laterite soil as an activator for persulfates in RhB decolorization studies. Natural laterite soil can be a potential substitute for chemical activators to avoid extensive utilization. Initially, the effect of raw laterite soil was investigated by holding the PS and the other parameters constant while gradually raising the concentration of raw laterite soil from a low concentration to a high concentration (50–200 mg L^−1^). The RhB decolorization was observed to increase as the laterite soil concentration was increased slowly (Figure 3). An increase in PS activation and subsequent rise in SO_4_^•−^OH^•^ and Fe (IV) ions could have accounted for the difference in decolorization. The rich content of iron in the laterite soil is an active agent for the activation of persulfate to SO_4_^•−^ generation and also, the presence of dissolved oxygen in the system leads to the formation of OH^•^ and Fe (IV) during the process. On a similar note, Nidheesh et al. [28] discussed the influence of laterite soil as a heterogenous E-Fenton source for the demineralization of mixed industrial effluent.

The higher decolorization was observed at raw laterite soil dose of 150 mg L^−1^. From these results, raw laterite soil as an environmentally friendly, easily accessible, and low-cost material, can be an alternative material for persulfate activation. However, because of the interference of other organic contamination of raw laterite soil, the high amount of raw laterite soil has a negative effect on the decolorization efficiency. The higher laterite concentration reduced the decolorization efficiency at 200 mg L^−1^ which can be attributed to the scavenging of HO^•^ by the intermediates [65].

**Figure 4:**
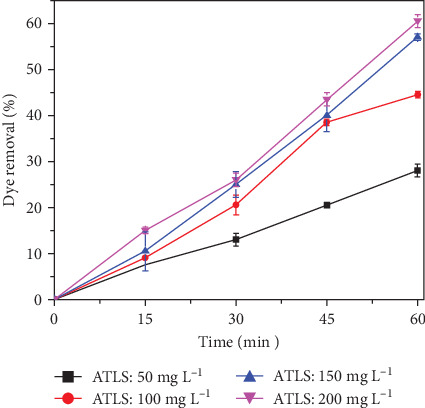
Effect of alkali-treated laterite soil (ATLS) dosage as a catalyst on persulfate activation for the decolorization of RhB (Experimental conditions: RhB: 150 mg L^−1^, persulfate dose 200 mg L^−1^, pH (7.00 ± 0.5)).

In order to circumvent the difficulties inherent in PS activation posed by raw laterite soil, additional research was conducted on laterite soil that had been treated with alkali.  Figure 4 clearly shows that the decolorization increases with increasing alkali-treated laterite soil (ATLS) dose. The increased generation of SO_4_^−•^ and Fe (IV) ions results in the decolorization of RhB. The catalyst doses of 150 and 200 mg L^−1^ exhibit the greatest decolorization (57% and 60.5%, respectively). The catalyst dose of 150 mg L^−1^ was chosen as the optimum dose for PS activation in the following process. Similar studies have shown promising results regarding the activation of persulfate using both raw and modified laterite soil for the treatment of dye wastewater. Raw laterite soil was able to reduce RhB concentrations by about 20% in 60 min, while ATLS achieved 57% to 60% removal in the same timeframe at pH 3, increasing to nearly complete removal after 72 h of biological treatment [43]. Additionally, biochar loaded with metal ions effectively activated persulfate and degraded various dyes, achieving removal rates of 41.1%–89.8% for four dyes and complete degradation under optimal conditions [66]. Furthermore, TiO_2_–Fe_3_O_4_ nanocomposites under UV–LED irradiation with persulfate showed approximately 98.1% decolorization efficiency and 61.1% mineralization of reactive red 198 under optimized conditions [67]. The efficiency of persulfate activation using laterite soil was reported as 91.4% for PAHs removal [68]. In contrast, the efficiency of persulfate activation using modified laterite soil was not explicitly mentioned in the provided contexts. However, the activation of persulfate using different methods such as heat, FeSO_4_, NaOH, and H_2_O_2_ showed removal efficiencies of 91.4%, 86.6%, 72.9%, and 86.2% for PAHs, respectively [68]. These results indicate that the choice of activation method significantly influences the efficiency of persulfate in contaminant degradation. The consecutive RhB degradation mechanism by alkaline laterite soil-based activation of persulfate oxidation is expressed in Equation (2).

Another important factor that can influence the rate of SO_4_^•−^ and Fe (IV) ions formation is pH. To investigate the effect of pH on PS activation, different pH conditions (pH: 3, 6, 7, 9, and 12 (±0.5)) were tested in conjunction with the other operating parameters. The concentrations of RhB: 150 mg L^−1^, PS: 200 mg L^−1^, and ATLS: 200 mg L^−1^ were kept constant.

(3) $$\begin{array}{c} S_{2}O_{8}^{2-}+\text{Rh}-\text{B}+\frac{{\text{Fe}}^{2+}-L}{\left(\text{Alkaline}\right)}\to {\text{SO}}_{4}^{•-}+{\text{SO}}_{4}^{2-}+{\text{CO}}_{2}+{\text{H}}_{2}O. \end{array}$$

**Figure 5:**
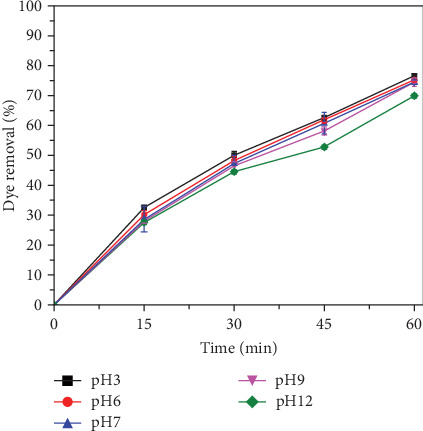
Reduction of RhB at different pH conditions. (Experimental conditions: RhB: 150 mg L^−1^, persulfate dose 150 mg L^−1^, alkali-treated laterite soil (ATLS): 200 mg L^−1^, and variable pH (3, 6, 7, 9, and 12 (±0.5)).

The results presented in Figure 5 depict a noticeable trend in COD removal from textile wastewater across varying pH conditions. Particularly, at pH 3, a significantly higher removal rate of 76.5% was observed, attributable to the favorable conditions for hydroxyl radical generation with a higher oxidation potential in an acidic environment [5, 37, 69]. The removal of dye reached approximately 75% at neutral pH conditions (6 and 7), with the maximum oxidation attributed to SO_4_^•−^ and ferryl ions generated during the process. This finding aligns with previous studies on textile wastewater treatment using PSOs activated by iron [5, 43].

Under near-neutral and alkaline conditions, ferrous ions exist in insoluble hydroxide forms like Fe(OH)^+^, Fe(OH)_2_, and Fe(OH)^3−^, rendering them inefficient for persulfate activation [5, 37, 69]. Thus, the activation of persulfate at higher pH conditions mainly occurs via cathodic reduction [5]. The pH level of the solution has a dual impact, influencing both the valence of iron and the reactions involving persulfate anions and pollutants. Despite a slower removal rate under alkaline pH conditions, the efficiency of COD removal after 60 min of electrolysis remains consistent across all tested pH conditions. In alkaline conditions, the process's efficiency is regulated by hydroxy radicals, produced through the interaction between SO_4_^•−^ and hydroxyl ions (Equation (1)) [70]. Additionally, outside of alkaline conditions, HO^•^ are generated in sulfate radical–based AOPs through the reaction between SO_4_^•−^ and water, as outlined in Equation (2) [71]. Under acidic conditions, the presence of iron-activated persulfate leads to the destruction of dye effluents, resulting in a faster COD degradation rate [72].

Equations (3) and (4) represent the reactions involved:

(4) $$\begin{array}{c} {\text{SO}}_{4}^{•-}+{\text{OH}}^{-}\to {\text{SO}}_{4}^{2-}+{\text{HO}}^{•}. \end{array}$$

(5) $$\begin{array}{c} {\text{SO}}_{4}^{•-}+H_{2}O\to {\text{SO}}_{4}^{2-}+{\text{HO}}^{•}+H^{+}. \end{array}$$

Comparisons were made with the previous results [73] of three different oxidation processes employed for textile wastewater treatment: UV/PS (persulfate), UV/H_2_O_2_, and UV/PMS (potassium monopersulfate). The UV/PS process, conducted under optimal conditions at pH 7, exhibited remarkable removal efficiencies with 85.5% COD removal, 65.7% TOC removal, and 99.0% color removal. The calculated COD/PS ratio of 1/5 showcased the efficiency of persulfate as a catalyst. Moreover, UV/PS exhibited the lowest total cost among the three processes, with an electricity cost of 23.9$/kg COD removed and a chemical usage cost of 4.33$/kg COD removed. In contrast, our study revealed a noticeable trend in COD removal under varying pH conditions. At pH 3, we observed a significantly higher removal rate of 76.5%, attributed to the favorable conditions for hydroxyl radical generation in an acidic environment. Furthermore, at neutral pH conditions (6 and 7), a COD removal efficiency of around 75% aligns with the findings from the UV/PS process, emphasizing the significance of SO_4_^•−^ and ferryl ions in oxidation processes activated by iron. In comparison to UV/H_2_O_2_, which exhibited optimal conditions at pH 4 and demonstrated competitive removal efficiencies, our study showcased consistent COD removal efficiency across all tested pH conditions. UV/PMS, while effective in color removal, faced limitations in organic matter removal and incurred the highest overall costs among the three processes.

Shakira et al. [43] reported that ATLS, acting as a catalyst, achieved a removal rate of 57%–60% for a model pollutant at pH 3. Yang et al. [74] demonstrated that the Fe (II)/KPS process exhibited greater effectiveness at pH 6 compared to pH 4, with the presence of protonated hydroxylamine further enhancing pollutant degradation. Furthermore, the activation of persulfate by iron complexes with picolinic acid was found to be effective within the pH range of 4.0–6.0 [74]. In highly alkaline conditions, oxidation is primarily caused by SO_4_^•−^ and ferryl ions. To confirm the effect of each oxidant at different pH levels, scavenging experiments were carried out to check the effect of the individual radical on dye degradation.

**Figure 6:**
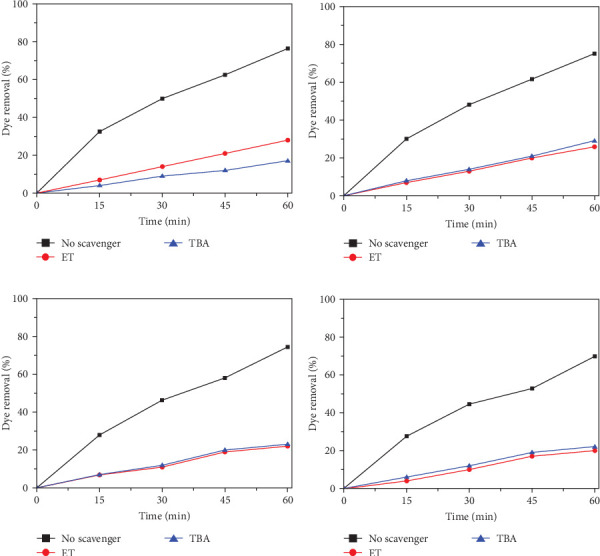
Reduction of RhB at different pH conditions. (Experimental conditions: RhB: 150 mg L^−1^, persulfate dose 150 mg L^−1^, alkali-treated laterite soil (ATLS): 200 mg L^−1^). The reduction of RhB at (a) pH 3; (b) pH 6; (c) pH 9; and (d) pH 12 in presence and absence of radical scavengers. ET, ethanol; TBA, tert-butyl alcohol.

During the experiments, tert-butyl alcohol (TBA) was used to scavenge HO^•^ and SO_4_^•−^, while ET was used to scavenge HO^•^ only. Before beginning the experiment, 15 mL of ET and 25 mL of TBA (0.25 N equivalent) were added to check for scavenging reactions. At the start of the experiment, the optimal amounts of persulfate and ATLS were also added. Using a UV–Visible spectrophotometer, samples were taken at regular intervals and residual dye concentrations were determined.  Figure 6a shows at pH 3, the contributions of the hydroxyl and SO_4_^•−^ are nearly equal.  Figure 6b,c shows that the sulfate radical contribution to dye degradation is substantially greater than the hydroxyl radical's.  Figure 6d demonstrates that the main causes of dye degradation are SO_4_^•−^ and ferrous ions. Very little dye degradation occurs in high alkali conditions, which is highly susceptible to hydroxyl ions.

### 3.4. Treatment of RhB by Combined Persulfate Oxidation Process Followed by Biological Treatment Process

The biological treatment method is particularly successful when it comes to the treatment of low concentrations of dye in industrial wastewater; however, when the dye concentration was increased, the degradation process took much longer (Figure 1). Similar results were also encountered in some previous evidence [75–78]. Shanumugam et al. [79] observed a similar problem where the higher dye concentration inhibits the biological process, which is overcome by diluting the dye concentration to ten times.

**Figure 7:**
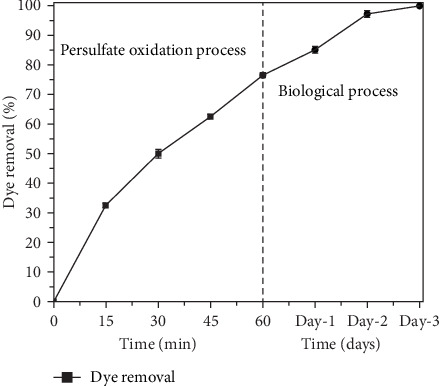
Reduction of RhB as a function of reaction time for combined persulfate oxidation processes (PSOs) followed by biological process. Persulfate oxidation process experimental conditions: persulfate dose 150 mg L^−1^, alkali-treated laterite soil (ATLS): 200 mg L^−1^, and variable pH (3 ± 0.5). (Biological process experimental conditions: RhB: 150 mg L^−1^, pH: 7.00 ± 0.5, and temperature: 30 ±2.0°C).

The biological degradation process was used after 60 min of PS oxidation process as shown in Figure 7. The residual dye concentration after the PS oxidation process was 35.25 mg L^−1^. When dye wastewater was treated further with the biological treatment process, almost all dye effluents (~99%) were removed after 72 h of treatment, despite high dye concentrations. The dye degradation efficiency is relatively less as compared with PS process.

### 3.5. Application on Real Filed Waters

The hybrid system's applicability, consisting of the persulafte oxidation process followed by the biological treatment process, was also evaluated in the real field water samples (dye effluents) obtained from Chirala, which is located in the Bapatal district of Andhra Pradesh in India. The initial characteristics of wastewater are detailed in Table 1, which may be found here.

**Figure 8:**
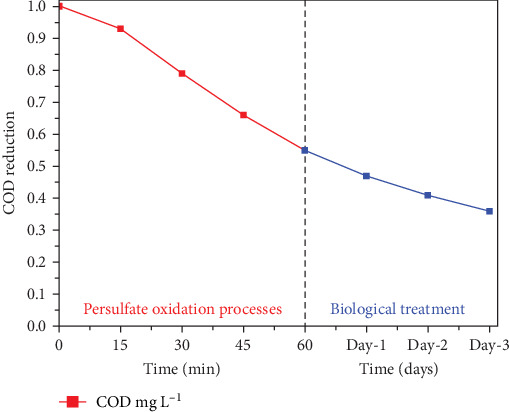
Reduction in chemical oxygen demand (COD) as a function of reaction time for combined PS process and biological process. (Experimental conditions persulfate oxidation process (PSO): COD: 1984 mg L^−1^, persulfate dose 150 mg L^−1^, alkali-treated laterite soil (ATLS): 200 mg L^−1^, and variable pH: 3 (±0.5). Biological: pH: 7 (±0.5) and temperature: 30 ± 2.0°C).

Water with a higher COD than tap water is mainly due to dyes being contaminated with water, which causes the COD to be higher. The experiments were conducted under optimal conditions and the results of the experiments are shown in Figure 8. During the oxidation of persulfate, the amount of COD was observed to be removed at approximately 45%. Upon further treatment with a biological oxidation process, the elimination effectiveness may increase to up to 64% in 3 days. During the initial PS process, it was seen that the highest COD was decreasing within the first 60 min of operation. This was a positive sign. Following the PS oxidation process, there is a subsequent improvement in the wastewater's biodegradability. PSO has the capability of oxidizing about more than 60% of the total organic contaminants that are found in wastewater. It has been noted that PSO, which is then followed by a biological treatment procedure, is an efficient treatment for the effluents produced by the textile industry. In addition to this, it was found that there was no accumulation of sludge during the procedure. Comparative studies are established in Table 2 to delineate the effectiveness of the current study with hybrid treatment mechanism.

The use of laterite soil and persulfate in the treatment process has both benefits and potential environmental impacts. Laterite soil is a natural, locally available material, making it a sustainable choice, but excessive use could lead to resource depletion. This can be managed by sourcing it responsibly and reusing spent material after regeneration. Persulfate is an effective oxidant, but its overuse can produce sulfate ions and reactive byproducts that might harm aquatic ecosystems. To address this, we carefully optimized the persulfate dosage and ensured proper neutralization of residues before discharge. Overall, the process balances effective treatment with sustainability and environmental safety.

## 4. Conclusion

In summary, the present investigation into the treatment of RhB dye in wastewater culminated in a multifaceted approach employing the PSO and biological treatment. The biological treatment effectively degraded RhB, with the anaerobic process achieving complete removal of 1 mg L^−1^ RhB in 4 days and longer durations for higher initial concentrations. The PSO process, with an optimal persulfate dose of 200 mg L^−1^, exhibited enhanced decolorization, particularly at concentrations of 200 and 250 mg L^−1^, with a decolorization efficiency of approximately 60%. The activation of persulfate using raw and ATLS demonstrated environmental and cost advantages, with alkali-treated soil exhibiting higher efficiency (60.5%). The influence of pH on persulfate activation revealed a significant COD removal trend, with a removal rate of 76.5% at pH 3 and approximately 75% at neutral pH (6 and 7). Integration of PSO with biological treatment successfully addressed higher dye concentrations, removing nearly 99% of dye effluents after 72 hr. Real field water testing confirmed the applicability of the hybrid system, showcasing notable improvements in physicochemical characteristics, including a 64% reduction in COD after the combined treatment. Comparisons with other oxidation processes highlighted the efficiency and cost-effectiveness of the proposed approach. Overall, our combined PSO and biological treatment emerged as a promising, sustainable, and adaptable solution for dye-containing wastewater, offering practical and environmentally conscious applications in the textile industry.

## 5. Future Research

Laterite soil is readily available in abundance in regions like Andhra Pradesh, Odisha, and other parts of India, and it is enriched with iron (Fe_2_O_3_). The results of this study provide valuable insights that can inform future research endeavors, particularly concerning the thermal activation of laterite soil as a pretreatment strategy to mitigate impurities. Laterite soil is characterized by its cost-effectiveness and sustainability, indicating its potential as an alternative catalyst in environmental applications. Its use may facilitate a significant reduction in the dependence on chemical-based iron dosages, thus, positioning laterite soil as a promising activating agent for AOPs. Continued investigation into these applications may further elucidate the roles of laterite soil in enhancing the efficiency and sustainability of treatment.

## Figures and Tables

**Table 1:** Physicochemical characteristics of dye industry effluent.

S. No.	Parameter	Initial	Biological (alone)	After PSO (alone)	After PSO + biological (hybrid)
1.	pH	7.8 (±0.5)	6.8 (±0.5)	3.0 (±0.5)	6.8 (±0.5)
2.	Turbidity (NTU)	288	176	130	61
3.	COD (mg L^−1^)	688.8	454.6	309.9	247.9
4.	BOD (mg L^−1^)	135	40	62	27
5.	TOC (mg L^−1^)	237	130	107	21
6.	TSS (mg L^−1^)	324	220	120	38
7.	TDS (mg L^−1^)	1842	1620	1521	543
8.	Chlorides (mg L^−1^)	1754	1240	812.25	654
9.	Sodium (mg L^−1^)	498.4	402.6	384	248
10.	Sulfates (mg L^−1^)	96.14	90.24	88.6	35

Abbreviations: BOD, biological oxygen demand; COD, chemical oxygen demand; PSO, persulfate oxidation process; TOC, total organic carbon.

**Table 2:** Comparative studies with other electrochemical treatments for Rhodamine-B decontamination.

S.No.	Process	Electrodes/catalyst	Operating conditions	Dye degradation	Reference
1	UV/H_2_O_2_	UV lamp = 250 nm, i–0.12 A, P_o/p_ = 6 W.	[Dye] = 10 μM; [H_2_O_2_] = 1.67 mM at pH 7, *T* = 25 ± 2°C	Color removal-73%	[80]

2	Humic acid—Fe@BioChar/PDS	—	—	99.76%	[81]

3	Photocatalysis	Cu–ZnO@ZrO_2_	—	98.4% in 14 min	[82]

4	Photo-Fenton	FeOCl—heterogeneous catalyst	Rh-B = 0.0104 mM, H_2_O_2_ = 6.52 mM, pH–3.6, *T* = 25°C, FeOCl–30 mg, 0.279 sun irradiance	95%	[83]

5	Photocatalysis adsorption	P-25 TiO_2_ (Degussa)	P-25 TiO_2_ = 0.65 g L^−1^, Rh-B = 20 mg L^−1^, UV = 352 nm, pH (range)−6–10	80%	[84]

6	Persulfate assisted AOPs	PS/CuO NPs	[RhB]_0_ = 50 mg L^−1^, [PS]_0_ = 1 mM, CuO = 0.5 g L^−1^ at pH = 7.5 and t = 45°C	> 90%	[85]

7	Anodic oxidation	Boron-doped diamond	pH = 7 (c) (Na_2_SO_4_) = 0.035 mol·dm^−3^, c (RhB) = 50 mg·dm^−3^, and t = 90 min, CE = 18.49%, and EEC was 44.58 kWh m^−3^	RhB = 98.58%, COD = 91.47%	[86]

8	Solar-Fenton process	ZnO enhanced solar Fenton	Fe^2+^:H_2_O_2_ = 1:3, RhB = 15 mg L^−1^, pH = 33.5, t – 30 min	RhB = 47%	[87]

9	Persulfate assisted AOPs	CA/nZVI/PS	RhB = 100 mg L^−1^, PS = 5 mmol·L^−1^, nZVI = 0.3 g·L^−1^, 0.1 mmol·L^−1^ CA, and pH of 5	RhB = 94.97%	[88]

10	RedMud assisted PS	RM/PS	pH 4.6, and RhB = 20 mg L^−1^, RM = 2 g L^−1^	RhB = 76.7%	[89]
	RMBC assisted PS	RM-grape fruit peel Biochar/PS	pH 4.6, and RhB = 20 mg L^−1^, RMBC = 2 g L^−1^	RhB = 89.98%	

11	Three-phase catalytic system enhanced persulfate oxidation	MnFe-LDH/PMS/O_3_	pH = 5.71, [MnFe-LDH]_0_ = 0.3 g L^−1^, [PMS]_0_ = 0.2 g L^−1^, [O_3_]_0_ = 0.3 L min^−1^	RhB = 98.3% in 12 min	[90]

12	S-scheme heterojunction photocatalysis	WS_2_/BiYWO_6_ Electrodes—Pt-counter, Ag/AgCl-reference, and sample-coated FTO	[RhB]_0_ = 10^−5^M	91% in 90 min	[91]

13	3-Dimensional electro-Fenton (3D/EF) system	Fe-Cu/Kaolin	pH = 3, Fe-Cu/kaolin particle = 30 g L^−1^, applied voltage = 10 V, aeration rate of 0.8 L min^−1^.	91.6%	[92]

14	Electro-Fenton	Cu_2_O/CNTs/PTFE (composite cathode)	pH = 3, CNTs:Cu_2_O: PTFE = 10:30:1	89.3% in 120 min	[93]

15	Biological treatment	Anaerobic batch reactor	[RhB]_0_ = 5 mg L^−1^	100% in 5 days	Current study
	PS/laterite soil	Persulfate oxidation	[RhB]_0_ = 150 mg L^−1^, PS = 150 mg L^−1^, ATLS = 200 mg L^−1^, pH = 3, t = 60 min	76.5% in 60 min	
16	Hybrid treatment	PS/ALTS/BT	[RhB]_0_ = 150 mg L^−1^, PS = 150 mg L^−1^, ATLS = 200 mg L^−1^, pH = 3, t = 60 min, (residual conc. PS = 35.25 mg L^−1^)	100% in 72 h	

Abbreviations: AOPs, advanced oxidation processes; ATLS, alkali-treated laterite soil; COD, chemical oxygen demand; PMS, peroxi mono sulfates.

## Data Availability

The data are available on request from the authors.
